# Association of Frontal Gray Matter Volume and Cerebral Perfusion in Heroin Addiction: A Multimodal Neuroimaging Study

**DOI:** 10.3389/fpsyt.2013.00135

**Published:** 2013-10-21

**Authors:** Niklaus Denier, André Schmidt, Hana Gerber, Otto Schmid, Anita Riecher-Rössler, Gerhard A. Wiesbeck, Christian G. Huber, Undine E. Lang, Ernst-Wilhelm Radue, Marc Walter, Stefan Borgwardt

**Affiliations:** ^1^Department of Psychiatry (UPK), University of Basel, Basel, Switzerland; ^2^Medical Image Analysis Centre, University Hospital Basel, Basel, Switzerland

**Keywords:** heroin addiction, biological parametric mapping, arterial spin labeling, voxel-based morphometry

## Abstract

Structure and function are closely related in the healthy human brain. In patients with chronic heroin exposure, brain imaging studies have identified long-lasting changes in gray matter (GM) volume. More recently, we showed that acute application of heroin in dependent patients results in hypoperfusion of fronto-temporal areas compared with the placebo condition. However, the relationship between structural and cerebral blood flow (CBF) changes in heroin addiction has not yet been investigated. Moreover, it is not known whether there is any interaction between the chronic structural changes and the short and long-term effects on perfusion caused by heroin. Using a double-blind, within-subject design, heroin or placebo (saline) was administered to 14 heroin-dependent patients from a stable heroin-assisted treatment program, in order to observe acute short-term effects. Arterial spin labeling (ASL) was used to calculate perfusion quantification maps in both treatment conditions, while Voxel-Based Morphometry (VBM) was conducted to calculate regional GM density. VBM and ASL data were used to calculate homologous correlation fields by Biological Parametric Mapping (BPM) and a whole-brain Pearson *r* correlation. We correlated each perfusion condition (heroin and placebo) separately with a VBM sample that was identical for the two treatment conditions. It was assumed that heroin-associated perfusion is manifested in short-term effects, while placebo-associated perfusion is more related to long-term effects. In order to restrict our analyses to fronto-temporal regions, we used an explicit mask for our analyses. Correlation analyses revealed a significant positive correlation in frontal areas between GM and both perfusion conditions (heroin and placebo). Heroin-associated perfusion was also negatively correlated with GM in the inferior temporal gyrus on both hemispheres. These findings indicate that, in heroin-dependent patients, low GM volume is positively associated with low perfusion within frontal regions.

## Introduction

Heroin dependence is associated with chronic exposure to heroin and is characterized by compulsive drug use despite negative consequences, including social drift (downward social mobility) (1). Cognitive processes, including impulse control (control of wishes and urges), are impaired after acute and chronic heroin exposure, as indicated by behavioral studies (2). Impaired impulse control in heroin addiction is reflected by imaging activity patterns that resemble those of immature brains (3, 4). These functionally abnormal activities with impulsive decision-making may be related to a relapse of heroin addiction. The compulsion to use heroin is frequently driven by enhanced stress responses and craving (desire for consumption) (5, 6), while acute heroin administration contributes to a reduction in these negative emotions, as indicated by reduced anxiety, craving, and stress hormone release (7–9). When heroin is administered, it crosses the blood brain barrier very rapidly in comparison to other opioids (10). Within the brain, the heroin metabolites 6-monoacetylmorphine (6-MAM) and morphine bind as agonists to μ and κ receptors (11). Both receptor types are found over the whole-brain (12, 13).

Structural magnetic resonance imaging (MRI) studies on the long-term effect of opioid application have demonstrated that opioid dependent subjects showed decreased gray matter (GM) in the bilateral prefrontal cortex and bilateral temporal cortex, including the insula (14). Furthermore, Yuan et al. (15) found that the GM of prefrontal, temporal, and insular regions was negatively correlated with the duration of heroin use, suggesting that heroin consumption has a cumulative effect.

Studies on cerebral blood flow (CBF) in heroin addiction have showed that chronic heroin use is associated with a decrease in global perfusion, with the greatest decrease in the frontal, occipital, and temporal regions (16). In our recently published study, we observed acute effects of heroin on cerebral perfusion (17). Our findings indicate that, in comparison to placebo, heroin leads to hypoperfusion in the left anterior cingulate cortex (ACC), the left medial prefrontal cortex (mPFC), and in bilateral insula.

However, the relationship between GM volume and perfusion in heroin addiction has not yet been investigated. In the current study, we examined whether there was an association between GM volume and perfusion in 15 heroin-addicted patients. Perfusion was assessed during the normal drug free condition (placebo) and after administration of heroin. We applied Biological Parametric Mapping (BPM) to assess the relation between perfusion – as measured by arterial spin labeling (ASL) – and GM volume – as measured by Voxel-Based Morphometry (VBM). The BPM toolbox was developed by Casanova and colleagues in MATLAB (18). This incorporates information obtained from other modalities as regressors for whole-brain analyses and also allows voxel-wise multimodal correlation. BPM has been used widely for integrative analysis of different neuroimaging modalities (19–21). On the basis of our previous findings (17), we expected a direct relationship between perfusion and GM within the areas with the most marked hypoperfusion during the acute effects of heroin. We hypothesized that, within the medial frontal and the temporal cortex, low perfusion should correlates with low GM volume.

## Materials and Methods

### Study sample

The study was approved by the local ethics committee and registered under http://clinicaltrials.gov (ID NCT01174927). After receiving a written and oral description of the study aims, all participants gave their written informed consent before inclusion.

Fourteen (eight male, six female; mean age 40.7 ± 6.8 years) non-left-handed heroin-dependent out-patients were recruited from the Center of Substance Use Disorders of the Department of Psychiatry in Basel University. The inclusion criteria were as follows: age older than 18 years, current heroin-maintained treatment for at least 6 months, with an unchanged heroin dose during the past 3 months. The exclusion criteria were a positive alcohol breathalyzer test, or an additional physical disease or psychiatric disorder other than substance dependencies. Clinically experienced psychiatrists conducted the Structured Clinical Interview for DSM-IV Axis II Disorders (SCID-II) (22) to assess the diagnosis of comorbid personality disorders.

Subjects reported their age of first heroin use (mean = 18.4 ± 2.9 years), years of heroin dependence (mean = 21.4 ± 7.2 years), duration of heroin maintenance (mean = 7.2 ± 3.9 years), and daily heroin dose (mean = 352 ± 178 mg). Patients were told to abstain from illicit drug use other than the prescribed heroin for the duration of the study, from alcohol intake for 72 h and from tobacco consumption for 2 h before scanning. Illicit substances and medications were controlled by a urine test at each session. Nevertheless, three patients were tested positive for cannabis and six patients for cocaine at one or both points of the measurement.

### Drug administration

In a cross-over, double-blind design, placebo (5 ml saline), and half of the daily heroin dose (mean = 176 ± 89.1 mg) were administered intravenously over a period of 30 s by a study nurse 20 min before the scanning session started. Heroin was dissolved in 5 ml of sterile water and aspirated into a syringe, according to the procedure described by Stohler et al. (23). Each patient was scanned twice, with a short interval between scans (mean 8.4 ± 3.3 days). On 1 day, patients received an injection of heroin or placebo (saline) before the scan and on the other day after the scan. Patients received their regular morning dose of heroin, corresponding to half of their daily individual dose.

### Image acquisition

Scanning was performed on a 3T MRI scanner (Magnetom Verio, Siemens Healthcare, Germany), using a 3D T1-weighted sequence (MPRAGE) for high resolution anatomical data and an ASL sequence (24) for quantification of CBF (perfusion). MPRAGE parameters were 1 mm × 1 mm × 1 mm isotropic resolution, repetition time of 2000 ms, inversion time of 1000 ms, and echo time of 3.4 ms. ASL was based on a flow-sensitive alternating inversion recovery spin labeling scheme (25), combined with modified Q2TIPS (TI periodic saturation) pulse preparation and a single-shot 3D gradient-spin echo readout (26). The sequence parameters were: repetition time 3200 ms, echo time 12.7 ms, and spatial resolution 4.6 mm × 4.6 mm × 4 mm (interpolated to 2.3 mm × 2.3 mm × 4 mm). Further details of ASL acquisition have been described elsewhere (17).

### Preprocessing of perfusion data

Cerebral blood flow (perfusion maps) was calculated by in-house software from ASL DICOM data. The difference images of the label and control images were first calculated and the time course was fitted to an ideal flow model. The equation for perfusion was then solved (24). The resulting perfusion maps were expressed in the unit ml/100 g/min. Conversion from the Metafile (MHD) to the NIFTI format was performed with MedINRIA Software^1^. Further preprocessing was conducted by Statistical Parametric Mapping (SPM8^2^), running under the MATLAB environment. Perfusion maps were first realigned and then masked with binarized intracranial tissue (binarization threshold: voxel intensity >0.1) to remove the extracerebral signal. Perfusion maps were then normalized to MNI space and smoothed using a 6 mm full-width-at half-maximum (FWHM) Gaussian kernel and proportionally scaled. More details of perfusion map preprocessing have been described elsewhere (17).

Voxel-Based Morphometry Structural data (MPRAGE) were preprocessed with VBM implemented in SPM8. MPRAGE images were non-rigidly normalized to a population-based average using diffeomorphic anatomical registration through exponentiated lie algebra (DARTEL) (27). The segmented tissue maps of GM were modulated with the Jacobian determinants from the spatial normalization to correct for volume changes. Finally, images were smoothed with an 8 mm FWHM Gaussian kernel.

Between condition differences were assessed by a voxel-wise whole-brain analysis using a general linear model (GLM). Statistical significance was assessed at cluster-level, using non-stationary random field theory (28). The first step of this cluster-level inference strategy consisted of identifying spatially contiguous voxels at a threshold of *p* < 0.01, without correction (cluster-forming threshold) (29). Finally, a family-wise error (FWE) corrected cluster-extent threshold of *p* < 0.05 was defined in order to infer statistical significance. A paired *t* test was used to examine the following contrasts: GM_heroin_ < GM_placebo_ and GM_heroin_ > GM_placebo_.

### Biological parametric mapping

**Figure 1:**
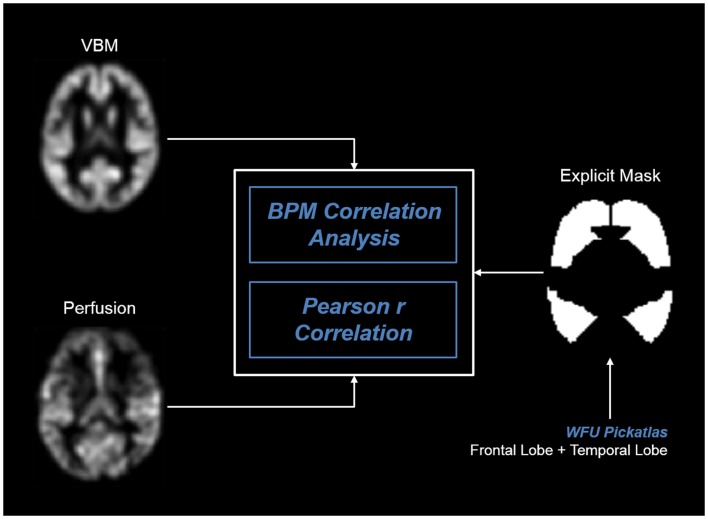
**BPM analysis pipeline of correlation analyses**.

For an integrative analysis of multimodal imaging data, we used BPM^3^ (18) running with SPM5 software. Preprocessed perfusion and VBM maps were realigned and used for whole-brain voxel-to-voxel correlation analysis. In order to restrict our analyses to fronto-temporal regions, that we had found to be hypoperfused during heroin (17), we used an explicit mask of the frontal and temporal lobe by WFU PickAtlas^4^. For overview, we refer to Figure 1.

In BPM, each perfusion condition (heroin and placebo) was correlated separately with the VBM data. Cluster-level inference was performed using a homologous correlation field (30) and a cluster-forming threshold of *p* < 0.01. Significant clusters were corrected for multiple comparisons with a FWE of *p* < 0.05 (29). MNI coordinates of significant clusters were converted into Talairach space and labeled with the Talairach Client 2.4.3^5^.

In addition, we calculated Pearson *r* correlations between perfusion and VBM data for each voxel within the standardized MNI space. We used the image calculation function implemented in SPM8 and applied the Pearson formula $r=\frac{\Sigma_{i}^{14}\left(x_{i}-\bar{x}\right)\left(y_{i}-ȳ\right)}{\sqrt{\Sigma_{i}^{14}\left(x_{i}-\bar{x}\right)\cdot \Sigma_{i}^{14}y_{i}-ȳ}}$ to our preprocessed images, where *x*_i_ and *y*_i_ represent perfusion and VBM values, and $\bar{x}$ and ȳ the mean perfusion and mean VBM values over the patient’s group, respectively (*n* = 14).

## Results

### Voxel-based morphometry analyses

Comparison of the heroin and placebo conditions found no significant difference in either direction (heroin > placebo and heroin < placebo).

### Multimodal correlation analyses

**Figure 2:**
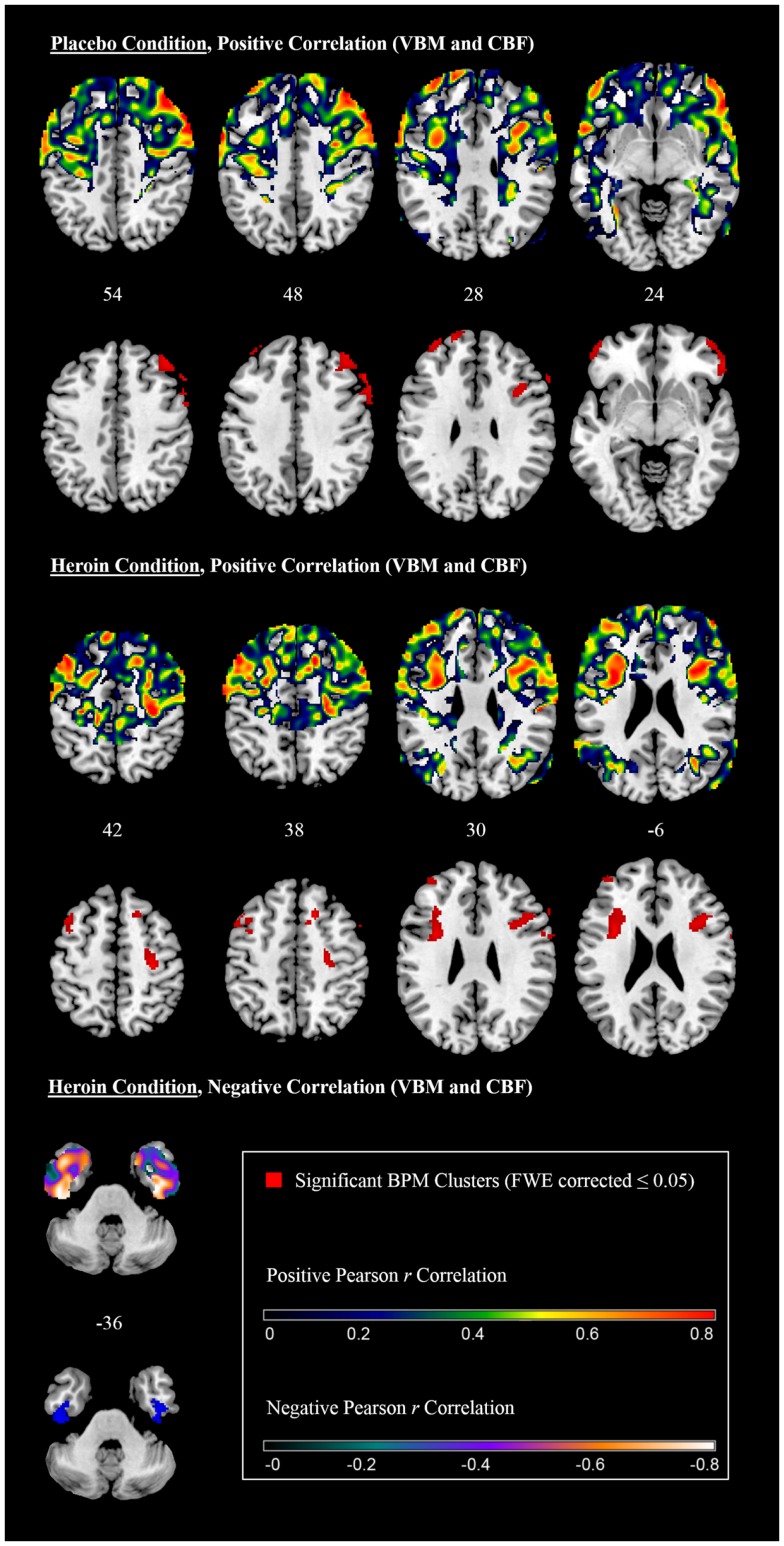
**Correlation between gray matter volume and perfusion (axial slices)**.

Biological Parametric Mapping analysis integrating GM and perfusion data after the placebo treatment revealed a significant positive correlation between perfusion and GM volume in frontal areas on both hemispheres including the precentral gyrus, the inferior, middle and superior frontal gyrus, the frontal pole, and the right paracingulate cortex, including parts of the ACC (Table 1 and Figure 2, upper panel). Pearson *r* values within the cluster maxima were all *r* > 0.7. No significant cluster was found in the temporal lobe and there was no significant negative correlation between modalities (Table 1).

**Table 1:** **Correlation between gray matter with placebo-associated perfusion**.

Area	Hemisphere	MNI coordinates of cluster maxima (*x, y, z*)	Cluster-size (voxels)	Cluster *p*-value (FWE corrected)	Pearson *r*
**POSITIVE CORRELATION**					
Precentral gyrus	R	60, 16, 40	544	<0.001	0.91
Inferior frontal gyrus	R	40, 16, 24			0.83
Middle frontal gyrus	R	58, 26, 36			0.81

Precentral gyrus	R	30, −22, 54	268	<0.001	0.93
Precentral gyrus	R	26, −16, 50			0.83
Precentral gyrus	R	42, −14, 62			0.78

Superior frontal gyrus	R	16, 22, 46	116	0.043	0.88
White matter	R	22, 22, 32			0.74
Paracingulate Gyrus/ACC	R	10, 14, 48			0.71

Precentral gyrus	L	−34, 10, 22	428	<0.001	0.91
Inferior frontal gyrus	L	−50, 8, 12			0.85
Middle frontal gyrus	L	−32, 24, 28			0.76

Middle frontal gyrus	L	−40, 20, 56	171	0.004	0.88
Middle frontal gyrus	L	−40, 6, 44			0.75
Middle frontal gyrus	L	−48, 22, 46			0.80

Frontal pole	L	−42, 54, 18	155	0.007	0.83
Frontal pole	L	−38, 52, 26			0.73
Frontal pole	L	−36, 46, 38			0.72

Precentral gyrus	R	22, −26, 74	121	0.034	0.80
**NEGATIVE CORRELATION**					
No significant clusters					

*L, left; R, right*.

Biological Parametric Mapping analysis integrating GM and perfusion data after the heroin treatment also showed a positive correlation between perfusion and GM volume in frontal areas. Significant clusters were found in the left precentral gyrus, the left middle and inferior frontal gyrus and the frontal pole on both hemispheres (Table 2 and Figure 2, middle panel). No significant cluster was found in the temporal lobe. Pearson *r* values within the cluster maxima were all *r* > 0.7. A negative correlation was found in the inferior temporal gyrus and the temporal fusiform cortex on both hemispheres (Table 2 and Figure 2, lower panel). Pearson *r* values within the cluster maxima were all less than −0.7.

**Table 2:** **Correlation between gray matter with heroin-associated perfusion**.

Area	Hemisphere	MNI coordinates of cluster maxima (*x, y, z*)	Cluster-size (voxels)	Cluster p-value (FWE corrected)	Pearson *r*
**POSITIVE CORRELATION**					
Precentral gyrus	R	60, 16, 40	166	0.008	0.92
Middle frontal gyrus	R	58, 26, 38			0.91
Middle frontal gyrus	R	52, 16, 50			0.80

Middle frontal gyrus	R	40, 40, 42	164	0.009	0.89

Inferior frontal gyrus	R	58, 36, −6	223	0.001	0.88
Frontal pole	R	50, 56, 2			0.86
Frontal pole	R	46, 56, −6			0.80
Inferior frontal gyrus	R	32, 14, 24	237	0.001	0.83
Middle frontal gyrus	R	40, 18, 32			0.80
Precentral gyrus	R	48, 6, 14			0.73
Frontal pole	L	−18, 58, 30	367	<0.001	0.83
Frontal pole	L	−40, 50, 26			0.81
Frontal pole	L	−50, 46, 6			0.78
**NEGATIVE CORRELATION**					
Inferior temporal gyrus	R	46 −8 −38	123	0.048	−0.80
Temporal fusiform cortex	R	40 −16 −36			−0.79

Inferior temporal gyrus	L	−46 −16 −34	149	0.016	−0.82
Temporal fusiform cortex	L	−38 −14 −32			−0.74

*L, left; R, right*.

## Discussion

In the present study, we examined the relationship between measurements of two different neuroimaging modalities in heroin-dependent patients, in particular between GM volumes and perfusion. We compared perfusion maps during acute heroin treatment and during placebo treatment with GM volumes by performing BPM correlations. We found that both placebo and heroin perfusion correlated positively with GM in frontal brain regions. Perfusion during placebo treatment, which we assumed to be related to the long-term effects of heroin, was also positively associated with GM in the cortical midline structure (ACC). We also found that heroin-associated perfusion correlated negatively with GM in the inferior temporal gyrus on both hemispheres. This negative correlation is difficult to explain in terms of our hypothesis that hypoperfusion is the driving force leading to GM reduction. The cuneus serves as visual processing and inhibitory control centers and electroencephalographic studies have revealed abnormalities in heroin-dependent individuals (31, 32) and decreased regional homogeneity (33) in this region. In our VBM analysis we found, as expected, no significant difference between heroin and placebo condition.

The regions showing a positive correlation between perfusion and GM are in line with regions showing reduced GM in heroin dependence (14, 15, 34, 35). Single photon emission computed tomography studies have also shown decreased perfusion in frontal and temporal cortex in opioid dependent patients (16, 36–38).

The frontal cortex is known to play an essential role in heroin addiction. In a recent study, we could show that heroin impairs stimulus-driven attention allocation, as indicated by reduced activity in the right inferior frontal gyrus (39). Further studies showed that loss of control over drug intake is not only a result of disrupted subcortical reward circuits but also of prefrontal higher-order executive functions (40). A recent study showed that prefrontal impairments in heroin addiction are directly associated with increases in impulsivity and the duration of heroin dependence (41). Studies with resting-state functional MRI in heroin addicts showed a decreased amplitude of low-frequency fluctuations in fronto-temporal regions (42), reduced regional homogeneity in the bilateral medial orbitofrontal cortex (33), and decreased functional connectivity in right dorsal ACC (43). Moreover, graph theoretical analysis demonstrated abnormal topological properties in areas of drug addiction-related circuits (44). Heroin addicts also showed altered default mode and rostral ACC network properties (45).

However, the question of how perfusion and GM interact in heroin dependence was not answered in our analysis. A possible explanation could be that heroin-associated perfusion in prefrontal and extended limbic areas may alter cerebral volume due to mild but chronic hypoxia and ischemia. This hypothesis is in line with animal models of brain ischemia, which showed that reduction in perfusion leads to neural death and cognitive impairment (46, 47). It is known that a heroin overdose can lead to coma with depressed respiration and increased pulse rate (48). These comas could cause a variety of neurological complications, including post-anoxic encephalopathy and cerebrovascular infarctions (49, 50). Interestingly, a study showed that GM differences between heroin addicts and a control group diminish after only 1 month heroin abstinence (51). This also supports the idea that GM deficits are caused by heroin itself and are not pre-existing before heroin addiction. The hypothesis that hypoperfusion leads to GM volume reduction by metabolic impairment, affecting neural, and glial function, is also in line with studies showing impaired white matter integrity in long-term addiction (52, 53). White matter microstructure deficits in heroin users are also associated with the duration of heroin dependence and impaired decision-making (54).

There are some limitations to this study, which should be carefully considered in interpreting our findings. Firstly, we only used a moderate number of subjects. A second limitation is the lack of a control group. It was thus not possible to analyze regional GM and perfusion differences with a between group approach and it therefore remains unclear how placebo-associated perfusion, reflecting normal perfusion in heroin-dependent patients, differs from that in healthy controls. A further difficulty is the aim of separating pure heroin effects on perfusion and structure from effects of other substances. Abstinence from tobacco use may induce craving, resulting in increased perfusion in areas associated with cigarette craving (55). However, all patients were smokers and therefore nicotine may be ruled out as confounding factor. In our previous analysis, we concluded that cocaine and cannabis did not significantly modify perfusion in our patient group (17).

In conclusion, we demonstrated that local GM volumes at baseline in heroin-dependent patients predicted perfusion in frontal regions after administration of placebo and heroin. These findings may suggest that recurrent and chronic hypoperfusion induced by heroin is a driving force for reduced GM in heroin addiction.

## Conflict of Interest Statement

The authors declare that the research was conducted in the absence of any commercial or financial relationships that could be construed as a potential conflict of interest.

## Footnotes

^1^http://www-sop.inria.fr/asclepios/software/MedINRIA/

^2^http://www.fil.ion.ucl.ac.uk/spm/

^3^http://www.nitrc.org/projects/rbpm/

^4^http://fmri.wfubmc.edu/software/pickatlas

^5^http://www.talairach.org/client.html
